# Ethnic differences in COVID-19 mortality during the first two waves of the Coronavirus Pandemic: a nationwide cohort study of 29 million adults in England

**DOI:** 10.1007/s10654-021-00765-1

**Published:** 2021-06-16

**Authors:** Vahé Nafilyan, Nazrul Islam, Rohini Mathur, Daniel Ayoubkhani, Amitava Banerjee, Myer Glickman, Ben Humberstone, Ian Diamond, Kamlesh Khunti

**Affiliations:** 1 Office for National Statistics, London, UK; 2 Nuffield Department of Population Health, Big Data Institute, University of Oxford, Oxford, UK; 3 London School of Hygiene and Tropical Medicine, London, UK; 4 Institute of Health Informatics, University College London, London, UK; 5 Diabetes Research Centre, University of Leicester, Leicester, UK

**Keywords:** COVID-19, Ethnicity, Mortality

## Abstract

Ethnic minorities have experienced disproportionate COVID-19 mortality rates in the UK and many other countries. We compared the differences in the risk of COVID-19 related death between ethnic groups in the first and second waves the of COVID-19 pandemic in England. We also investigated whether the factors explaining differences in COVID-19 death between ethnic groups changed between the two waves. Using data from the Office for National Statistics Public Health Data Asset, a linked dataset combining the 2011 Census with primary care and hospital records and death registrations, we conducted an observational cohort study to examine differences in the risk of death involving COVID-19 between ethnic groups in the first wave (from 24th January 2020 until 31st August 2020) and ﻿the first part of the second wave (from 1st September to 28th December 2020). We estimated age-standardised mortality rates (ASMR) in the two waves stratified by ethnic groups and sex. We also estimated hazard ratios (HRs) for ethnic-minority groups compared with the White British population, adjusted for geographical factors, socio-demographic characteristics, and pre-pandemic health conditions. The study population included over 28.9 million individuals aged 30–100 years living in private households. In the first wave, all ethnic minority groups had a higher risk of COVID-19 related death compared to the White British population. In the second wave, the risk of COVID-19 death remained elevated for people from Pakistani (ASMR: 339.9 [95% CI: 303.7–376.2] and 166.8 [141.7–191.9] deaths per 100,000 population in men and women) and Bangladeshi (318.7 [247.4–390.1] and 127.1 [91.1–171.3] in men and women) background but not for people from Black ethnic groups. Adjustment for geographical factors explained a large proportion of the differences in COVID-19 mortality in the first wave but not in the second wave. Despite an attenuation of the elevated risk of COVID-19 mortality after adjusting for sociodemographic characteristics and health status, the risk was substantially higher in people from Bangladeshi and Pakistani background in both the first and the second waves. Between the first and second waves of the pandemic, the reduction in the difference in COVID-19 mortality between people from Black ethnic background and people from the White British group shows that ethnic inequalities in COVID-19 mortality can be addressed. The continued higher rate of mortality in people from Bangladeshi and Pakistani background is alarming and requires focused public health campaign and policy changes.

**Supplementary Information:**

The online version contains supplementary material available at 10.1007/s10654-021-00765-1.

## Introduction

A recent systematic review of 50 studies have showed that people from ethnic minority background in the UK and other countries, particularly Black and South Asian groups, have been disproportionately affected by the Coronavirus (COVID-19) pandemic compared to people of White ethnic background [1] While several studies have investigated whether adjusting for socio-demographic and economic factors and medical history reduces the estimated difference in risk of mortality and hospitalisation [2–4], the reasons for the differences in the risk of experiencing harms from COVID-19 are still being explored during the course of the pandemic. Factors including structural racism [5, 6], social vulnerability [7, 8] social and material deprivation, [9] have widely been suggested as potential mechanisms for these reported inequalities.

In view of changes in policy, treatments and roll out of vaccination programmes, understanding the evolving nature of the COVID-19 epidemiology is crucial in helping shape the public health response to the coronavirus pandemic, especially in the context of emerging variants in some countries [10]. As emerging evidence suggest that the long-term consequences of COVID-19 may be severe, especially amongst people from ethnic minority groups [11], it is critical to monitor how ethnic inequalities throughout the course of the pandemic have evolved.

Using nationwide population-level data containing detailed socio-demographic characteristics and information on pre-pandemic health status, we compared the difference in risk of COVID-19 related death between ethnic groups in the two waves of the COVID-19 pandemic. We also investigated whether the factors explaining differences in COVID-19 death between ethnic groups changed between the two waves. To our knowledge, it is the first study to examine how the difference in the COVID-19 mortality between ethnic groups changed when adjusting for both detailed socio-demographic factors and pre-pandemic health at a whole population level.

## Methods

### Data

Using data from the Office of National Statistics (ONS) Public Health Data Asset on approximately 29 million adults aged 30–100 years living in private households in England, we conducted an observational cohort study to examine the differences in the risk of death involving COVID-19 between ethnic groups in the first wave (from 24th January 2020 until 31st August 2020) and the first part of the second wave (from 1st September to 28th December 2020) of the pandemic. Since data on socio-demographic factors are very scarce the healthcare datasets, we obtained these data from the 2011 Census. The 2011 Census was linked to the General Practice Extraction Service Data for Pandemic Planning and Research (GDPPR) which contains primary care records for all individuals living in England in November 2019. This dataset was further linked to mortality records, Hospital Episode Statistics, using the NHS number. To obtain NHS numbers for the 2011 Census, the 2011 Census was linked to the 2011–2013 NHS Patient Registers. It was first linked deterministically using 24 different matching keys, based on a combination of forename, surname, date of birth, sex and geography (postcode or Unique Property Reference Number). Probabilistic matching was then used to attempt to match records that were not linked deterministically, using 13 different combinations of personal identifiers. Candidate matches were assigned to Census records using the Felligi-Sunter probabilistic matching method. Of the 53,483,502 Census records, 50,019,451 were linked deterministically. 555,291 additional matches were obtained using probabilistic matching (overall linkage rate: 94.6%).

Of the 39,375,536 people enumerated at 2011 Census in England and Wales, aged 21–91 in 2011 (and would be 30–100 in 2020), we excluded 1,820,251 people (4.6%) who could not be linked deterministically or probabilistically to the NHS Patient register, and. 3,859,999 individuals (10.3%) who had died between the Census and 24th January 2020. An additional 4,400,447 people (13.1%) were not linked to the English primary care records because they either did not live in England in 2019 (the Census included people living in England and Wales), or were not registered with the NHS (see sample flow diagram in Supplementary Table A2). We restricted our analysis to people aged 30 to 100 in 2020 because most socio-demographic factors were drawn from the 2011 Census, and therefore may not represent people’s circumstances at the beginning of the pandemic younger people were thought particularly likely to have changed their circumstances. In addition, very few deaths occurred in people aged below 30 years: Official figures show that out of the 84,449 people who died from COVID-19 in 2020, only 127 (0.15%) were less than 30 years old [12]

### Outcomes

The outcome was COVID-19 related death (either in hospital or out of hospital), defined as confirmed or suspected COVID-19 death as identified by ICD-10 codes U07.1 or U07.2 mentioned on the death certificate anywhere on the death certificate. We analysed deaths in two time periods based on the death of occurrence: 24th January 2020 to 31st August 2020 (wave 1) and 1st September 2020 to 28th December 2020 (wave 2). We used 1st September as a cut-off date because the number of COVID-19 related death reached its lowest point in the week commencing 31^st^ August 2020 [12].

### Exposure

The exposure of interest was self-reported ethnicity obtained from the 2011 Census. We used a 10-category classification [13] and used the White British ethnic group as the reference category in all models. Ethnicity was imputed in 3.0% of 2011 Census returns due to item non-response using nearest-neighbour donor imputation, the methodology employed by the Office for National Statistics across all 2011 Census variables.

### Covariates

Other covariates used in the regression models include geographical factors (region, population density, Rural urban classification),socio-demographic characteristics (age, sex, index of multiple deprivation, housing, household composition, occupational exposure), and pre-pandemic health status (body mass index (BMI), learning disability, cancer, and immunosuppression, and other health conditions). Geographical factors were based on the 2019 Patient Register; socio-demographic characteristics were obtained from the 2011 Census (since this is the most reliable source for these variables); BMI and comorbidities were derived based on the primary care and hospitalisation data and defined using the QCOVID risk prediction model [14]. Details of these variables are available in Table 1.

We hypothesised that each of these factors may be associated with the risk of COVID-19 mortality by either increasing the risk of becoming infected and/or the risk of mortality once infected with COVID-19.

**Table 1:** Covariates included in the Cox-regression models

Variable	Coding
*Age variables*	
Single year of age	Second-order polynomial
*Geographical variables*	
Region	Dummy variables representing region of residence within England (South East, London, North West, East of England, West Midlands, South West, Yorkshire and the Humber, East Midlands, North East)
Population density of lower super output area (see table note)	Second-order polynomial, allowing for a different slope beyond the 99th percentile of the distribution to account for extreme values
Rural urban classification	Rural hamlets and isolated dwellings, Rural hamlets and isolated dwellings in a sparse setting, Rural town and fringe, Rural town and fringe in a sparse setting, Rural village, Rural village in a sparse setting, Urban city and town, Urban city and town in a sparse setting, Urban major conurbation, Urban minor conurbation
*Socio-economic variables*	
Index of multiple deprivation (IMD))	Dummy variables representing deciles of deprivation – from 1 (most deprived) to 10 (least deprived)
Household deprivation (see table note)	Not deprived, deprived in one dimension, deprived in two dimensions, deprived in three dimensions, deprived in four dimensions
Household tenure	Own outright, own with mortgage, social rented, private rented, other
Approximate social grade of the household reference person (see table note)	AB Higher and intermediate managerial/administrative/professional, C1 Supervisory, clerical, junior managerial/administrative/professional, C2 Skilled manual workers, D Semi-skilled and unskilled manual workers, E On state benefit, unemployed, lowest grade workers (based on household tenure for people aged 75 or over)
Level of highest qualification	Degree, A-level or equivalent, GCSE or equivalent, no qualification
*Household variables*	
Household size	1–2 people, 3–4 people, 5–6 people, 7 + people
Multigenerational household	Dummy for households with at least one person 65 + and someone at least 20 years younger
Household with children	At least one child aged 9 to 18
*Occupational exposure variables (see table note)*	
Key worker type	Education & childcare, food & necessity goods, health & social care, public services, national & local government, public safety & national security, transport, utilities & communication, not a key worker
Key worker in the household	Yes, no
Exposure to disease	Score ranging from 0 (no exposure) to 100 (maximum exposure), derived from O*NET data [15]
Proximity to others	Score ranging from 0 (no exposure) to 100 (maximum exposure), derived from O*NET data [15]
Household exposure to disease	Maximum ‘exposure to disease’ score within each household
Household proximity to others	Maximum of ‘proximity to others’ score within each household
*Health-related variables*	
Body Mass Index (kg/m^2^)	< 18.5, 18.5 – 25, 25 to 30, > = 30, missing
Chronic kidney disease (CKD)	No CKD, CKD3, CKD4, CKD5
Learning disability	No learning disability, Down’s Syndrome, other learning disability
Cancer and immunosuppression	Dummies for blood cancer, solid organ transplant, Prescribed immunosuppressant medication by GP, Prescribed leukotriene or long-acting beta blockers, Prescribed regular prednisolone,
Other conditions	Diabetes, Chronic obstructive pulmonary disease (COPD), Asthma, Rare pulmonary diseases, Pulmonary hypertension or pulmonary fibrosis, Coronary heart disease, Stroke, Atrial Fibrillation, Congestive cardiac failure, Venous thromboembolism, Peripheral vascular disease, Congenital heart disease, Dementia, Parkinson’s disease, Epilepsy, Rare neurological conditions, Cerebral palsy, Severe mental illness (bipolar disorder, schizophrenia, severe depression), Osteoporotic fracture, Rheumatoid arthritis or Systemic lupus erythematosus, Cirrhosis of the liver

There are 32,844 Lower Super Output Area (LSOA) areas in England, with a mean population of 1500 and a minimum of 1000. We calculated density as LSOA population divided by LSOA area. Household deprivation is defined according to four dimensions: employment (at least one household member is unemployed or long-term sick, excluding full-time students); education (no household members have at least Level 2 education, and no one aged 16–18 years is a full-time student); health and disability (at least one household member reported their health as being ‘bad’/ ‘very bad’ or has a long-term health problem); and housing (the household’s accommodation is overcrowded, with an occupancy rating -1 or less, or is in a shared dwelling, or has no central heating). Approximate Social Grade is a socio-economic classification based the occupation, employment status, qualification, tenure and whether they work full time, part time or not working of the household reference person. Key worker type is defined based on the occupation and industry code. ‘Exposure to disease’ and ‘proximity to others’ are derived from the O*NET database, which collects a range of information about individuals’ working conditions and day-to-day tasks of their job. To calculate the proximity and exposure measures, the questions asked were: i) How physically close to other people are you when you perform your current job? ii) How often does your current job require that you be exposed to diseases or infection? Scores ranging from 0 (no exposure) to 100 (maximum exposure) were calculated based on these questions using methods previously described by the ONS

### Statistical analyses

As a measure of differences in absolute risk of COVID-19 mortality, we calculated age-standardized mortality rates (ASMRs) for the different ethnic groups, whereby the age distribution within each group was standardized to the 2013 European Standardised Population. We calculated ASMRs separately for men and women.

The differences in the risk of COVID-19-related death across ethnic groups could be mediated by geographical factors, socio-demographic characteristics and pre-pandemic health. These factors fall on the causal path between ethnicity and COVID-19 mortality in a directed acyclic graph. To assess whether these factors accounted for some of the difference in risk between ethnic groups, we estimated Cox’s proportional hazards models adjusted for a range of factors. First, we estimated models that only adjusted for age. The age-adjusted hazard ratios (HRs) can be interpreted as a measure of inequality in COVID-19 mortality. We then added groups of control variables (geographical factors, socio-demographic characteristics, and pre-pandemic health) step by step and assessed how these affected the estimated HRs. When fitting the Cox models, we included all individuals who died during the analysis period and a weighted random sample of those who did not, with a sampling rate of 1% for those of white British ethnicity and 10% for adults from ethnic minority groups.

Our primary analyses were restricted to people living in the community because the drivers of infections (and hence mortality) are likely to be different for people living in private household than for people living in communal establishments, including care homes. However, to examine the robustness of our primary findings we also calculated ASRMs by sex and ethnic group for the whole population including people living in communal establishments.

## Results

### Characteristics of the study population

Our analytical sample consisted of 28,946,702 people aged 30–100 years who were alive on 24 January 2020 and living in England in private households. The number of COVID-19 related deaths was 29,303 and 17,487 in the first (24th January 2020 to 31st August 2020) and ﻿the first part of the second wave (1st September 2020 to 28th December 2020) of the pandemic, respectively (Table 2).

In this cohort of people living in private households, 53% were women and the average age was 56 (SD: 16) years. 83% percent of individuals identified as people from the White British ethnic group. The gender and age distribution of those who had a COVID-19 related death was similar in the two periods. In the first period, women accounted for 40.8% of COVID-19 related death, and the mean age at death was 79(12) years. In the second period, women accounted for 41.4% of COVID-19 related death and the mean age at death was 79 (11) years. The mean age at death remained similar in the two waves for all ethnic group (See Supplementary Table A1). A higher proportion of COVID-19 related death occurred amongst people from White British ethnic background in wave 2 (87.6%) compared to wave 1 (83.6%), while the proportion of death decreased from 1.4% in wave 1 to 0.4% in wave 2 among people from Black African ethnic group, and 2.4% to 0.9% among people from Black Caribbean ethnic background. The proportion of deaths increased with the level of index of multiple deprivation deciles (Table 2).

**Table 2:** Demographic and medical characteristics for the study cohort and those who died with COVID-19 in the two waves

		Cohort	Deaths in wave 1	Deaths in wave 2
Age	Mean (SD)	56.12 (15.68)	79.14 (11.58)	79.31 (10.88)
Sex	Male	13,652,990 (47.17)	17,350 (59.21)	10,243 (58.57)
	Female	15,293,712 (52.83)	11,953 (40.79)	7244 (41.43)
Ethnicity	Bangladeshi	186,199 (0.64)	204 (0.70)	157 (0.90)
	Black African	395,746 (1.37)	423 (1.44)	61 (0.35)
	Black Caribbean	310,759 (1.07)	702 (2.40)	156 (0.89)
	Chinese	154,724 (0.53)	100 (0.34)	34 (0.19)
	Indian	787,033 (2.72)	915 (3.12)	473 (2.70)
	Mixed	341,909 (1.18)	200 (0.68)	76 (0.43)
	Other	666,895 (2.30)	646 (2.20)	196 (1.12)
	Pakistani	507,626 (1.75)	546 (1.86)	587 (3.36)
	White British	24,066,373 (83.14)	24,483 (83.55)	15,312 (87.56)
	White other	1,529,438 (5.28)	1,084 (3.70)	435 (2.49)
Urban rural classification	Rural hamlets and isolated dwellings	930,665 (3.22)	622 (2.12)	339 (1.94)
	Rural hamlets and isolated dwellings in a sparse setting	84,000 (0.29)	44 (0.15)	38 (0.22)
	Rural town and fringe	2,562,682 (8.85)	2378 (8.12)	1383 (7.91)
	Rural town and fringe in a sparse setting	108,796 (0.38)	96 (0.33)	60 (0.34)
	Rural village	1,611,199 (5.57)	1,244 (4.25)	679 (3.88)
	Rural village in a sparse setting	95,815 (0.33)	77 (0.26)	59 (0.34)
	Urban city and town	12,716,134 (43.93)	11,375 (38.82)	6933 (39.65)
	Urban city and town in a sparse setting	51,759 (0.18)	41 (0.14)	31 (0.18)
	Urban major conurbation	9,731,718 (33.62)	12,285 (41.92)	6795 (38.86)
	Urban minor conurbation	1,053,934 (3.64)	1,141 (3.89)	1,170 (6.69)
Population density	Mean (SD)	4340.85 (4512.37)	4599.51 (4584.24)	4038.8 (3742.77)
Household deprivation	Not deprived	14,176,524 (48.97)	5,078 (17.33)	2,680 (15.33)
	Deprived in 1 dimension	9,054,362 (31.28)	11,294 (38.54)	6518 (37.27)
	Deprived in 2 dimensions	4,325,112 (14.94)	10,479 (35.76)	6760 (38.66)
	Deprived in 3 dimensions	1,266,548 (4.38)	2251 (7.68)	1427 (8.16)
	Deprived in 4 dimensions	124,156 (0.43)	201 (0.69)	102 (0.58)
IMD decile	1 (most deprived)	2,566,911 (8.87)	3298 (11.25)	2570 (14.70)
	2	2,690,016 (9.29)	3291 (11.23)	2148 (12.28)
	3	2,798,502 (9.67)	3140 (10.72)	1939 (11.09)
	4	2,877,203 (9.94)	2976 (10.16)	1790 (10.24)
	5	2,945,882 (10.18)	2812 (9.60)	1648 (9.42)
	6	2,976,122 (10.28)	2901 (9.90)	1581 (9.04)
	7	3,018,386 (10.43)	2817 (9.61)	1627 (9.30)
	8	3,031,461 (10.47)	2698 (9.21)	1514 (8.66)
	9	3,039,238 (10.50)	2707 (9.24)	1471 (8.41)
	10 (least deprived)	3,002,981 (10.37)	2663 (9.09)	1199 (6.86)
Approximate social grade	AB	6,600,071 (22.80)	3944 (13.46)	1949 (11.15)
	C1	8,596,874 (29.70)	7758 (26.48)	4306 (24.62)
	C2	6,313,753 (21.81)	5741 (19.59)	3617 (20.68)
	D	6,478,963 (22.38)	9592 (32.73)	6232 (35.64)
	E	957,041 (3.31)	2268 (7.74)	1383 (7.91)
Highest educational attainment	No qualification	5,705,728 (19.71)	14,907 (50.87)	9775 (55.90)
	Level 1	4,013,069 (13.86)	1948 (6.65)	1140 (6.52)
	Level 2	4,250,387 (14.68)	2,318 (7.91)	1,210 (6.92)
	Apprenticeship	1,064,673 (3.68)	1812 (6.18)	1152 (6.59)
	Level 3	3,445,156 (11.90)	1405 (4.79)	789 (4.51)
	Level 4 +	8,875,463 (30.66)	4824 (16.46)	2255 (12.90)
	Other	1,592,226 (5.50)	2089 (7.13)	1166 (6.67)
Household tenancy	Owned outright	8,490,537 (29.33)	16,160 (55.15)	9787 (55.97)
	Owned with a mortgage	11,921,447 (41.18)	4392 (14.99)	2569 (14.69)
	Shared ownership	212,921 (0.74)	169 (0.58)	80 (0.46)
	Social rented (from council)	2,116,854 (7.31)	3560 (12.15)	2179 (12.46)
	Social rented (other)	1,820,542 (6.29)	2873 (9.80)	1658 (9.48)
	Private rented	4,123,099 (14.24)	1672 (5.71)	924 (5.28)
	Living rent free	261,302 (0.90)	477 (1.63)	290 (1.66)
Type of accommodation	Detached house	7,530,682 (26.02)	6712 (22.91)	3925 (22.45)
	Semi-detached house	9,776,779 (33.78)	10,465 (35.71)	6,864 (39.25)
	Terraced	7,290,579 (25.19)	6875 (23.46)	4259 (24.36)
	Flat (purposed built)	3,179,138 (10.98)	4457 (15.21)	2085 (11.92)
	Flat (converted)	861,580 (2.98)	521 (1.78)	175 (1.00)
	Flat (Commercial building)	225,105 (0.78)	114 (0.39)	65 (0.37)
	Other	82,839 (0.29)	159 (0.54)	114 (0.65)
Household size	1–2	17,303,404 (59.78)	24,489 (83.57)	14,677 (83.93)
	3–4	10,058,379 (34.75)	3897 (13.30)	2229 (12.75)
	5 +	1,403,614 (4.85)	747 (2.55)	432 (2.47)
	Missing	181,305 (0.63)	170 (0.58)	149 (0.85)
Multigenerational household		3,393,523 (11.72)	4471 (15.26)	2707 (15.48)
Household with children		6,185,983 (21.37)	1,124 (3.84)	710 (4.06)
Overcrowded household		2,362,797 (8.16)	1,704 (5.82)	787 (4.50)
Key worker	Education and childcare	1,788,153 (6.18)	1,043 (3.56)	603 (3.45)
	Food and necessary goods	202,322 (0.70)	287 (0.98)	170 (0.97)
	Health and social care	2,124,226 (7.34)	1576 (5.38)	896 (5.12)
	Key public services	455,962 (1.58)	323 (1.10)	176 (1.01)
	National and Local Government	225,341 (0.78)	227 (0.77)	119 (0.68)
	Not keyworker	23,038,882 (79.59)	24,924 (85.06)	14,997 (85.76)
	Public safety and national security	395,003 (1.36)	309 (1.05)	167 (0.95)
	Transport	331,906 (1.15)	393 (1.34)	241 (1.38)
	Utilities and communication	384,907 (1.33)	221 (0.75)	118 (0.67)
Proximity to other	Mean (SD)	58.77 (19.62)	57.44 (19.54)	57.41 (19.21)
Exposure to disease	Mean (SD)	19.13 (20.96)	17.34 (19.43)	16.82 (18.68)
Key worker in household		10,105,744 (34.91)	7409 (25.28)	4176 (23.88)
BMI	< 18.5	260,872 (0.90)	852 (2.91)	376 (2.15)
	18.5 to 25	5,499,789 (19.00)	5915 (20.19)	2998 (17.14)
	25 to 30	6,107,438 (21.10)	6261 (21.37)	3663 (20.95)
	> = 30	5,204,914 (17.98)	6510 (22.22)	4027 (23.03)
	Missing	11,873,689 (41.02)	9765 (33.32)	6423 (36.73)
Chronic Kidney disease	None	28,457,417 (98.31)	26,354 (89.94)	15,609 (89.26)
	CDK 3	423,973 (1.46)	2040 (6.96)	1369 (7.83)
	CDK 4	43,593 (0.15)	544 (1.86)	364 (2.08)
	CDK 5	21,719 (0.08)	365 (1.25)	145 (0.83)
Learning disability	No	28,647,716 (98.97)	27,889 (95.17)	16,786 (95.99)
	Learning disability	291,322 (1.01)	1380 (4.71)	690 (3.95)
	Down’s syndrome	7,664 (0.03)	34 (0.12)	11 (0.06)
Cancer and immunosuppression	Blood cancer	323,011 (1.12)	1197 (4.08)	677 (3.87)
	Respiratory cancer	8,792 (0.03)	161 (0.55)	51 (0.29)
	Taking immunosuppressants	7,081 (0.02)	33 (0.11)	24 (0.14)
	Taking anti-leukotriene or long acting beta2-agonists	2,186,147 (7.55)	5839 (19.93)	4008 (22.92)
	Taking oral steroids in the last 6 months	385,167 (1.33)	2531 (8.64)	1492 (8.53)
Other comorbidities	Cerebral Palsy	3,870 (0.01)	36 (0.12)	10 (0.06)
	Asthma	3,401,127 (11.75)	3998 (13.64)	2550 (14.58)
	Atrial Fibrillation	1,055,408 (3.65)	6129 (20.92)	3748 (21.43)
	Coronary heart disease	1,512,855 (5.23)	6875 (23.46)	4628 (26.47)
	COPD	1,031,712 (3.56)	4576 (15.62)	3245 (18.56)
	Cystic fibrosis or bronchiectasis or alveolitis	356,141 (1.23)	2,023 (6.90)	1074 (6.14)
	Dementia	298,106 (1.03)	5758 (19.65)	2647 (15.14)
	Diabetes	2,970,375 (10.26)	9819 (33.51)	5840 (33.40)
	Epilepsy	312,184 (1.08)	682 (2.33)	362 (2.07)
	Heart failure	523,438 (1.81)	4462 (15.23)	2799 (16.01)
	Liver cirrhosis	79,379 (0.27)	309 (1.05)	195 (1.12)
	Neurological disease	25,335 (0.09)	152 (0.52)	91 (0.52)
	Parkinson’s disease	103,103 (0.36)	981 (3.35)	495 (2.83)
	Peripheral vascular disease	294,850 (1.02)	1913 (6.53)	1286 (7.35)
	fracture of hip, wrist, spine or humerus	27,197 (0.09)	195 (0.67)	121 (0.69)
	Pulmonary hypertension or fibrosis	123,176 (0.43)	1477 (5.04)	776 (4.44)
	Rheumatoid arthritis or SLE	306,581 (1.06)	875 (2.99)	540 (3.09)
	Severe mental illness	5,645,703 (19.50)	5322 (18.16)	3,196 (18.28)
	Stroke or TIA	849,332 (2.93)	5078 (17.33)	2821 (16.13)
	Thrombosis or pulmonary embolus	6,862 (0.02)	42 (0.14)	26 (0.15)

Linked 2011 Census to HES, GDPPR and Mortality registration data. Sample restricted to people living in private households

### Differences in COVID-19 mortality in wave 1 and wave 2: Age-standardized mortality rates

Table 3 shows the age-standardized mortality rates (ASMR) by ethnic group separately for the first and the second waves of the pandemic. In the first wave, the ASMRs of COVID-19 mortality were greatest among individuals identifying as Black African (402.5 [95% CI 341.6–463.4] and 174.4 [CI 137.6–210.5] deaths per 100,000 population in men and women, respectively). The ASMRs were lowest among those identifying as White British (119.1 [117.1–121.1] and 65.1 [63.8–66.3] deaths per 100,000 population in men and women, respectively). Levels of absolute risk were greater among all ethnic-minority groups compared with the White British population.

**Table 3:** Age standardised mortality rates (ASMRs) of death involving COVID-19 per 100,000 population, stratified by sex and ethnic group

	Wave 1 (24th Jan 2020–31st Aug 2020)		Wave 2 (1st Sep 2020–28th Dec 2020)	
	Women	Men	Women	Men
Bangladeshi	153.9 (112.1–204.6)	378.2 (307.0–449.3)	127.1 (91.1–171.3)	318.7 (247.4–390.1)
Black African	174.1 (137.6–210.5)	402.5 (341.6–463.4)	32.0 (17.6–51.6)	79.7 (45.0–124.2)
Black Caribbean	146.2 (127.1–165.2)	348.2 (314.1–382.4)	35.6 (26.9–46.1)	79.7 (63.3–98.7)
Chinese	82.9 (57.3–115.6)	155.6 (116.4–202.9)	44.0 (24.9–71.6)	43.7 (24.9–70.8)
Indian	120.3 (106.7–133.9)	236.9 (216.6–257.3)	64.6 (54.5–74.6)	124.2 (109.0–139.3)
Mixed	99.6 (76.8–126.6)	220.4 (179.2–261.6)	48.2 (32.6–68.4)	75.0 (52.0–103.9)
Other	124.0 (106.5–141.6)	246.4 (219.6–273.3)	52.5 (41.1–66.1)	83.3 (65.9–100.7)
Pakistani	157.1 (133.0–181.2)	281.7 (249.7–313.7)	166.8 (141.7–191.9)	339.9 (303.7–376.2)
White British	65.1 (63.8–66.3)	119.1 (117.1–121.1)	42.6 (41.5–43.6)	77.8 (76.1–79.4)
White other	66.4 (60.2–72.7)	155.0 (142.7–167.3)	28.3 (24.1–32.5)	65.2 (57.0–73.5)

The ASMRs were standardised to the 2013 European Standardised population. 95% confidence intervals of the ASMRs in parentheses

In the second wave, the ASMRs of COVID-19 mortality were highest among men and women identifying as Pakistani (339.9 [303.7–376.2] and 166.8 [141.7–191.9] deaths per 100,000 population in men and women) and Bangladeshi (318.7 [247.4–390.1] and 127.1 [91.1–171.3] deaths per 100,000 population in men and women) ethnic background. The ASMRs of COVID-19 mortality were lowest for people from other White background (65.2 [57.0–73.5] and 28.3 [24.1–32.5] deaths per 100,000 population in men and women) and the White British population (65.2 [57.0–73.5] and 28.3 [24.1–32.5] deaths per 100,000 population in men and women). Unlike in the first period, the ASMRs of COVID-19 mortality for people from Black African and Black Caribbean were similar to the ASMRs for people from the White British group.

ASMRs of COVID-19 mortality for all residents, including people living in the communal establishments (e.g., care homes) are higher, especially in the first wave for people of White British background. However, the ethnic differences remained similar to those observed for people living in private household (Supplementary Table A3).

### Determinants of disparities in COVID-19 mortality between ethnic groups

**Fig. 1:**
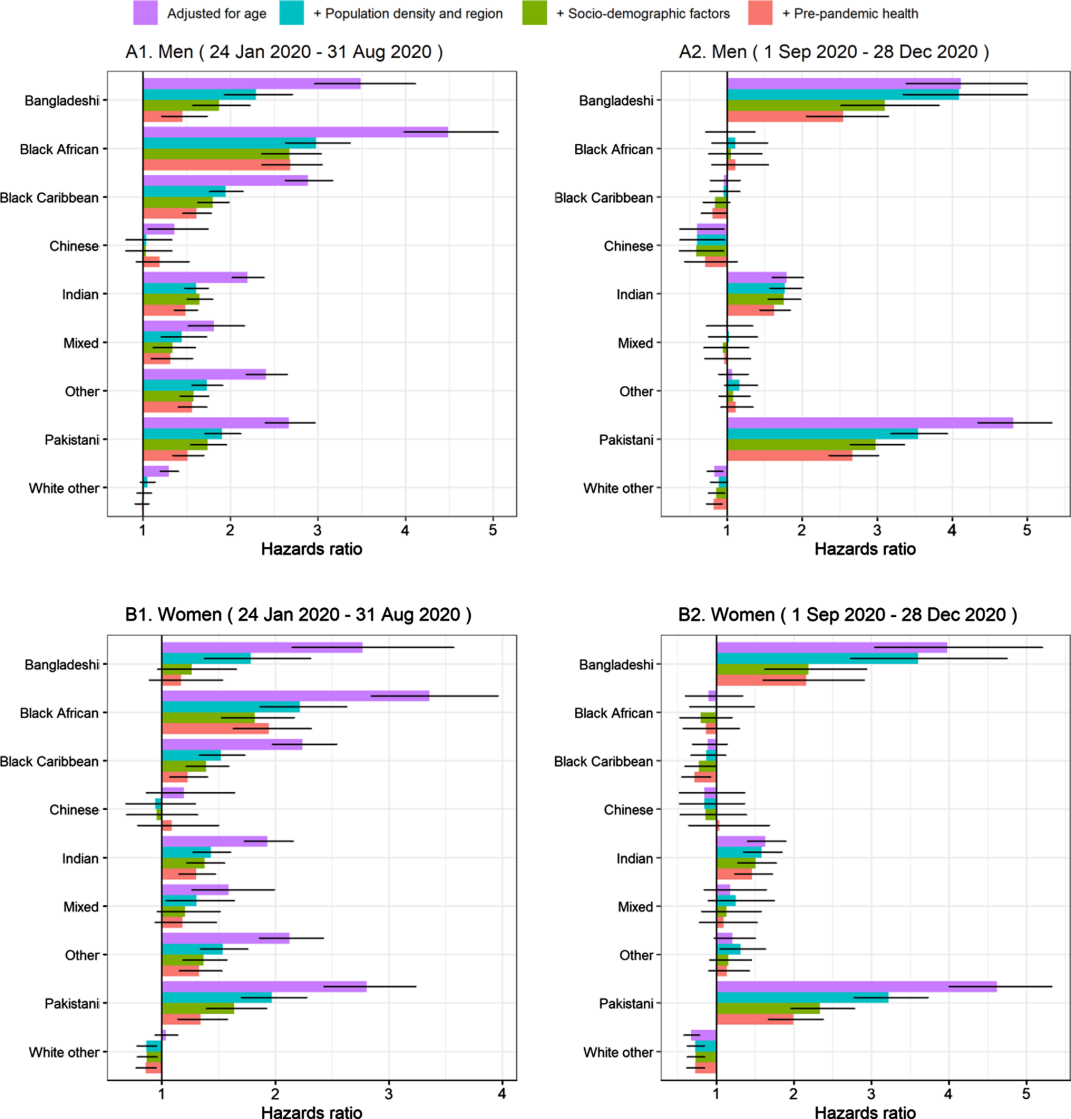
Hazard ratios for COVID-19 related death for ethnic-minority groups compared with the White British population, stratified by sex and pandemic waves. *Note* Results obtained from Cox-regression models. Geographical factors: dummies for region of residence, for urban/rural classification and second order polynomial of population density of Lower Super Output Area (LSOA). Socio-demographic characteristics include Index of Multiple Deprivation (IMD), household deprivation, household tenure, social grade, level of highest qualification, household size, multigenerational household, household with children, key worker type, key worker in the household, exposure to disease, proximity to others, household exposure to disease, household proximity to others. Pre-pandemic health include Body Mass Index (kg/m^2^), Chronic kidney disease (CKD), Learning disability, Cancer and immunosuppression, other conditions (See Supplementary Tables A1 for more details). Numerical results can be found in Supplementary Tables A4)

Figure 1 reports hazard ratios (HR) of COVID-19 related death in the first wave and second wave in men and women for ethnic minority groups compared with the White British population.

As indicated by the ASMRs, age-adjusted HRs indicated that men and women from all ethnic-minority groups (except women of Chinese and White Other ethnicity) were at greater risk of COVID-19 related death compared with those of White British ethnicity in the first wave. The highest risk of mortality was observed among people from Black African ethnic background. For example, compared with men from White ethnic background, the rate of COVID-19 related deaths in wave 1 was 4.49 (95% confidence interval [CI]: 3.98–5.07) times higher in men from Black African ethnicity. In wave 2, men and women from South Asian ethnic groups were at greater risk of death involving COVID-19 compared with those of White British ethnicity (Fig. 1), with adjusted HRs of 4.81 [4.34–5.32] and 4.62 [4.01–5.33] in men and women from Pakistani background, and 4.11 [3.38–4.99] and 3.98 [3.04 -5.20] in men and women from Bangladeshi background, respectively. Individuals from Indian background also had elevated risk of COVID-19 related death, with adjusted HRs of 1.80 [1.60–2.01] and 1.63 [1.40–1.90] in men and women, respectively. Unlike in wave 1, people from Black ethnic groups were not at greater risk of COVID-19 death compared to those of White British ethnicity.

In both waves, adjusting for geographical factors, socio-demographic characteristics and pre-pandemic health substantially reduced the estimated disparities between most ethnic groups and the White British population. This suggests that the differences in mortality between ethnic groups are partly mediated by these factors. However, these factors attenuated the hazard ratios more strongly in the first than in the second wave. In addition, the factors that most strongly affected the HRs differed in the two waves.

In the first wave, adjusting for geographical factors more than halved the estimated hazard ratios for all ethnic minority groups. For most groups, the hazard ratios were further reduced by adjusting for socio-demographic factors and pre-pandemic health status, especially amongst women. After adjusting for all these factors, women from Bangladeshi and Mixed background were no longer at greater risk of COVID-19 related death. For women from all other groups except Black African, the fully adjusted hazard ratios were below 1.4. However, despite the attenuation of the hazard ratios after full adjustment, men from all ethnic minority groups but other White remained at greater risk, but with hazard ratios greatly attenuated.

In the first part of the second wave, adjusting for geographical factors did not substantially reduce the HRs in men and women from Bangladeshi background, but attenuated the HRs for people from Pakistani background. Adjusting for socio-demographic factors attenuated the elevated risks of people from Bangladeshi and Pakistani background similarly in the two waves. Further adjustment for pre-pandemic health status also attenuated the relationship. However, even after full adjustment, people from Pakistani and Bangladeshi background remained substantially at greater risk of COVID-19 deaths than White British people, with HRs of 2.67 [2.36 –3.02] and 1.99 [1.67—2.38]in men and women from Pakistani background, and 2.55 [2.06—3.15] and 2.16 [1.60–2.91] in men and women from Bangladeshi background, respectively. The adjustments had little impact on the HRs for people from Indian background.

## Discussion

### Summary of findings

In this analysis of 28.9 million adults living in private households in England and 46,790 COVID-19 related deaths, we highlight several major findings. First, in the first wave of the COVID-19 pandemic all ethnic minority groups were at elevated risk of COVID-19 related death. In the second wave, people from South Asian background, in particular Bangladeshi and Pakistani, but not Black individuals, were at greater risk of COVID-19 death compared to the White British population. Second, geographical factors explained more than half of the differences in COVID-19 mortality risk in the first wave, but much less in the second wave. Third, socio-demographic factors explained a similar proportion of the elevated risks of people from Bangladeshi and Pakistani background in the first and second waves. Fourth, adjusting for comorbidities did not substantially reduce the ethnic difference in risk of COVID-19 related death, after other factors that had already been accounted for.

### Comparison with related studies

In line with existing studies investigating ethnic inequalities in SARS-CoV-2 infection and COVID-19 mortality [3, 4, 16–18], we find that most ethnic minority groups were disproportionally affected in the first wave. Our findings that the ethnic inequalities in COVID-19 mortality differed between the two waves is consistent the evidence that these disparities are likely to be driven by differences in exposure to infection and therefore can change over time. Existing evidence suggests that the lockdown measures implemented in March 2020 were associated with a reduction in inequalities in mortality in England in all ethnic minority groups [3]. Our results are also consistent with a recent study of clinical records for 40% patients in England showing that the ethnic differences in the risk of severe outcomes changed in the second wave [4].

Several studies analysed the ethnic inequalities in COVID-19 mortality in the first wave, adjusting for detailed socio-demographic factors [3] or detailed pre-existing health conditions [4]. Our study is the first to investigate simultaneously the role of socio-demographic factors and health conditions in explaining the differences in COVID-19 mortality between ethnic groups between the first and the second wave in a large nationwide population. We find that after adjusting for geographical and socio-demographic factors, adjusting for pre-existing conditions only moderately reduced the estimated differences in COVID-19 mortality between ethnic groups. This suggests that these inequalities in mortality are primarily driven by differences in exposure and infection, which is corroborated by findings from a study based on antibody testing [18].

### Strengths and limitations

The primary strength of our study is the use of a unique, nationwide, newly linked population-level data set based on the General Practice Extraction Service (GDPPR) Data for pandemic planning and research, linked to the most comprehensive and reliable sources of sociodemographic variables from the latest census, mortality records and Hospital Episode Statistics. Unlike studies based solely on electronic health records, our study is based on self-identified ethnicity, with very few missing data, limiting the potential for exposure misclassification bias Our data contain both detailed socio-demographic characteristics, such as household composition, housing quality, and occupational exposure, and extensive information on pre-pandemic health based on primary care and hospital records. To our knowledge, our study is the first to use nationally representative linked data to examine the association between ethnicity and COVID-19 mortality while accounting for the effect of both socio-demographic factors and comorbidities.

The main limitation of our study data set is the 9-year lag between census day and the start of the pandemic. Most socio-demographic characteristics included in our models reflect the situations of individuals as they were in 2011, not necessarily those at the start of the COVID-19 pandemic. To mitigate this, we excluded people aged less than 30 years old, whose circumstances are the most likely to have changed since the Census. We also updated place of residence based on information from the 2019 NHS Patient Register. Since the socio-demographic factors are less likely to have changed for older people than younger people, measurement error is likely to be smaller for the people at greater risk. Some measurement error is nonetheless likely to reduce the explanatory power of the socio-demographic factors and pre-existing conditions included in the model, thereby reducing their effect on the hazard ratios. In addition, the outcome variable, COVID-19-related death, may be measured with an error, as not all COVID-19-related deaths may have been captured on death certificates. Conversely, not all deaths for which COVID-19 was mentioned on the death certificate may have involved the disease. There is no reason to believe that these potential outcome misclassifications differ between ethnic, therefore this is unlikely to bias the estimated hazard ratios, but may reduce the precision. Another limitation is that the study population is limited to people enumerated at the 2011 Census, and therefore did not include people who immigrated or were born between 2011 and 2020. As a result, it did not fully represent the population at risk. However, migrants tend to be young and the risk of COVID-19 mortality is low for young people [12].

### Mechanisms

We find that in the second wave the disparities are more pronounced in people of South Asian ethnicity particularly those from Pakistani and Bangladeshi backgrounds. Compared to people from other ethnic groups, these groups are more likely to reside in deprived areas, in large households and in multigenerational families [3]. Households are important contributor to transmission of COVID-19, with household size being associated with risk of SARS-CoV-2 infection [19–21]. Secondary attack rates within household are high [22], and as a result living in multi-generational household is associated with increased risk of COVID-19 mortality amongst elderly adults in England [23]. Differences in occupational exposure could also account for some of the differences in mortality between groups, as a higher proportion of Pakistani and Bangladeshi men work as taxi drivers, shopkeepers and proprietors than any other ethnic backgrounds [24]. Previous research showed that ethnic minority groups also experience other structural factors that increase their likelihood of risk of mortality [25].

Whilst our study adjusts for a range of socio-demographic factors, including household composition and occupational exposure, we may not capture fully the effect of these factors because of measurement error. Our study also accounts for differences in pre-pandemic health. Potential contributing factors not measured in our data include linguistic and cultural factors as well as barriers to accessing public health messaging [26]. Further research, including qualitative studies, would be needed to understand better the differences observed between the waves.

### Implications of the findings

The finding of a strong reduction in the difference in COVID-19 mortality between people from Black ethnic background and people from the White British group is reassuring. The widespread coverage in national media of research findings and government reports published during the first wave of infection that highlighted that people form ethnic minority groups were disproportionally affected by COVID-19 may have helped raise the awareness of these disparities amongst the general public. This raised awareness may have led to behavioural changes that may have reduced infection and mortality amongst people from Black ethnic background. However, the continued higher rate of mortality in people from Bangladeshi and Pakistani background is alarming, and requires focused public health campaign and policy response. Focusing on treating underlying conditions, although important, may not be enough to reduce the inequalities in COVID-19 mortality. Understanding the need of these ethnic groups, through engagement with local communities, public health and healthcare teams, must be at the core of any public health response.

## Conclusion

Our study showed that the risk of COVID-19 mortality during the first wave of COVID-19 pandemic was higher in people from ethnic minority background, both in men and women, compared to people from White ethnic background. There was a reduction of COVID-19 mortality during the second wave in most of the ethnic groups while the higher rates continued in men and women from Bangladeshi and Pakistani background. Focused public health policy may help reduce the existing and widening inequalities in COVID-19 mortality.

## Supplementary Information

Below is the link to the electronic supplementary material.

Supplementary file1 (DOCX 73 kb)

## Declarations

### Data sharing statement

The ONS Public Health Linked Data Asset will be made available on the ONS Secure Research Service for Accredited researchers. Researchers can apply for accreditation through the Research Accreditation Service. The data will include all variables used in this analysis.
