# Supplementary material for: Ethnic differences in COVID-19 mortality during the first two waves of the Coronavirus Pandemic: a nationwide cohort study of 29 million adults in England
Source: Eur J Epidemiol. 2021 Jun 16;36(6):605–17. doi: 10.1007/s10654-021-00765-1 (PMC8206182; doi:10.1007/s10654-021-00765-1)
Supplement: Supplementary file 1 — Supplementary file1 (DOCX 73 kb) [file 10654_2021_765_MOESM1_ESM.docx]

### Supplementary Tables

Table A2 Sample selection and number of participants

| **Inclusion criteria** | **Cohort size** |
| --- | --- |
| All enumerated at 2011 Census in England and Wales, aged 21-91 in 2011* | 39,375,536 |
| Linked to the NHS 2011-2013 Patient Register | 37,555,285 |
| Alive on 24th January 2020 | 33,695,286 |
| Linked to 2019 English primary care records | 29,294,839 |
| Living in private households | 28,946,702 |

*These people would be 30 to 100 in 2020

Table A2 Mean age at death by ethnic group in the two waves


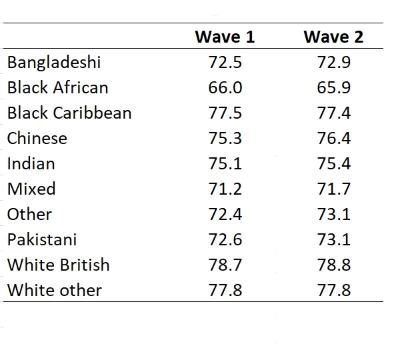


Note: Linked 2011 Census to HES, GDPPR and Mortality registration data. Sample restricted to individuals 30-100 years old living in private households

Table A3 Age standardised mortality rates (ASMRs) of death involving COVID-19 per 100,000 population, stratified by sex and ethnic group, including people living in communal establishments

|  |  |  |  |  |
| --- | --- | --- | --- | --- |
|  | Wave 1 (24th Jan 2020 - 31st Aug 2020) | | Wave 2 (1st Sep 2020 - 28th Dec 2020) | |
|  | Females | Males | Females | Males |
| Bangladeshi | 162.8 (120.1 - 214.4) | 381.9 (310.7 - 453.2) | 126.7 (90.9 - 170.6) | 317.9 (246.7 - 389) |
| Black African | 197.3 (158.5 - 236.2) | 447.4 (381.5 - 513.3) | 31.4 (17.4 - 50.5) | 82.3 (47.7 - 126.1) |
| Black Caribbean | 182.2 (161.4 - 203) | 397 (361.2 - 432.8) | 38.9 (29.8 - 49.7) | 86.2 (68.4 - 103.9) |
| Chinese | 122.1 (90.8 - 160.4) | 193.3 (149.4 - 245.3) | 52.6 (31.8 - 81.6) | 49.6 (29.3 - 78.3) |
| Indian | 147.5 (132.4 - 162.5) | 259.4 (238 - 280.8) | 68.2 (57.9 - 78.5) | 127.3 (111.9 - 142.7) |
| Mixed | 142.4 (114.3 - 170.5) | 249.4 (206.4 - 292.5) | 53.3 (37.2 - 73.6) | 77.4 (54.5 - 106.1) |
| Other | 151.1 (131.8 - 170.5) | 276.1 (247.4 - 304.7) | 53.6 (42.2 - 66.9) | 87 (69.5 - 104.5) |
| Pakistani | 165.3 (140.5 - 190.1) | 290.7 (258.1 - 323.2) | 167.7 (142.6 - 192.8) | 339.7 (303.5 - 375.9) |
| White British | 94 (92.6 - 95.5) | 152.6 (150.4 - 154.8) | 50.8 (49.7 - 51.9) | 85.5 (83.8 - 87.2) |
| White other | 96.5 (89.2 - 103.8) | 197.4 (183.7 - 211) | 34.8 (30.2 - 39.3) | 72.4 (63.7 - 81) |

Note: The ASMRs were standardised to the 2013 European Standardised population. 95% confidence intervals of the ASMRs in parentheses

Table A4 Hazard ratios for COVID-19 related death for ethnic-minority groups compared with the White British population stratified by sex and period

|  |  |  |  |  |
| --- | --- | --- | --- | --- |
|  | Age adjusted | + geographical factors | + Socio-dem. | + health status |
|  | A. Men - Wave 1 (24th Jan 2020 - 31st Aug 2020) | | | |
| Bangladeshi | 3.489 (2.960 - 4.112) | 2.288 (1.933 - 2.708) | 1.866 (1.566 - 2.222) | 2.519 (2.035 - 3.117) |
| Black African | 4.489 (3.984 - 5.057) | 2.976 (2.630 - 3.367) | 2.674 (2.355 - 3.035) | 2.680 (2.360 - 3.044) |
| Black Caribbean | 2.883 (2.626 - 3.165) | 1.942 (1.761 - 2.142) | 1.794 (1.625 - 1.980) | 1.609 (1.456 - 1.778) |
| Chinese | 1.358 (1.058 - 1.742) | 1.035 (0.806 - 1.329) | 1.034 (0.804 - 1.329) | 1.188 (0.923 - 1.528) |
| Indian | 2.194 (2.021 - 2.382) | 1.605 (1.474 - 1.747) | 1.646 (1.507 - 1.797) | 1.485 (1.359 - 1.624) |
| Mixed | 1.810 (1.516 - 2.160) | 1.444 (1.208 - 1.725) | 1.336 (1.118 - 1.596) | 1.309 (1.095 - 1.564) |
| Other | 2.401 (2.178 - 2.648) | 1.726 (1.561 - 1.909) | 1.578 (1.423 - 1.751) | 1.559 (1.405 - 1.730) |
| Pakistani | 2.666 (2.399 - 2.963) | 1.901 (1.707 - 2.117) | 1.739 (1.549 - 1.951) | 1.509 (1.342 - 1.696) |
| White other | 1.296 (1.198 - 1.402) | 1.049 (0.968 - 1.137) | 1.012 (0.933 - 1.097) | 0.985 (0.908 - 1.068) |
|  | B. Men - Wave 2 (1st Sep 2020 - 28th December) | | | |
| Bangladeshi | 4.111 (3.384 - 4.993) | 4.090 (3.348 - 4.998) | 3.101 (2.515 - 3.824) | 2.547 (2.059 - 3.150) |
| Black African | 0.990 (0.716 - 1.368) | 1.111 (0.801 - 1.540) | 1.053 (0.758 - 1.463) | 1.112 (0.800 - 1.546) |
| Black Caribbean | 0.959 (0.783 - 1.176) | 0.950 (0.773 - 1.169) | 0.842 (0.684 - 1.036) | 0.807 (0.655 - 0.995) |
| Chinese | 0.596 (0.371 - 0.960) | 0.601 (0.373 - 0.968) | 0.591 (0.367 - 0.953) | 0.704 (0.437 - 1.136) |
| Indian | 1.795 (1.600 - 2.014) | 1.767 (1.570 - 1.989) | 1.751 (1.549 - 1.980) | 1.627 (1.437 - 1.841) |
| Mixed | 0.985 (0.725 - 1.339) | 1.029 (0.756 - 1.399) | 0.945 (0.695 - 1.286) | 0.965 (0.709 - 1.313) |
| Other | 1.066 (0.886 - 1.283) | 1.166 (0.966 - 1.407) | 1.080 (0.892 - 1.308) | 1.112 (0.919 - 1.347) |
| Pakistani | 4.809 (4.344 - 5.323) | 3.540 (3.184 - 3.937) | 2.979 (2.641 - 3.360) | 2.668 (2.360 - 3.016) |
| White other | 0.832 (0.735 - 0.941) | 0.887 (0.783 - 1.006) | 0.852 (0.751 - 0.967) | 0.822 (0.725 - 0.933) |
|  | C. Women - Wave 1 (24th Jan 2020 - 31st Aug 2020) | | | |
| Bangladeshi | 2.769 (2.147 - 3.570) | 1.783 (1.377 - 2.309) | 1.263 (0.963 - 1.656) | 1.169 (0.891 - 1.534) |
| Black African | 3.356 (2.844 - 3.961) | 2.215 (1.867 - 2.629) | 1.818 (1.525 - 2.167) | 1.943 (1.630 - 2.317) |
| Black Caribbean | 2.239 (1.973 - 2.540) | 1.518 (1.331 - 1.731) | 1.389 (1.216 - 1.587) | 1.226 (1.072 - 1.402) |
| Chinese | 1.193 (0.868 - 1.641) | 0.941 (0.683 - 1.295) | 0.954 (0.692 - 1.314) | 1.089 (0.790 - 1.501) |
| Indian | 1.929 (1.726 - 2.156) | 1.431 (1.277 - 1.604) | 1.376 (1.221 - 1.551) | 1.301 (1.152 - 1.469) |
| Mixed | 1.588 (1.267 - 1.991) | 1.303 (1.039 - 1.636) | 1.204 (0.959 - 1.512) | 1.180 (0.940 - 1.481) |
| Other | 2.124 (1.862 - 2.422) | 1.535 (1.340 - 1.757) | 1.367 (1.189 - 1.572) | 1.329 (1.156 - 1.529) |
| Pakistani | 2.804 (2.430 - 3.236) | 1.967 (1.700 - 2.275) | 1.636 (1.392 - 1.922) | 1.341 (1.141 - 1.576) |
| White other | 1.036 (0.941 - 1.140) | 0.864 (0.783 - 0.953) | 0.867 (0.785 - 0.957) | 0.857 (0.776 - 0.947) |
|  | D. Women - Wave 2 (1st Sep 2020 - 28th December) | | | |
| Bangladeshi | 3.979 (3.043 - 5.204) | 3.603 (2.735 - 4.747) | 2.186 (1.625 - 2.941) | 2.160 (1.604 - 2.908) |
| Black African | 0.897 (0.600 - 1.340) | 0.989 (0.659 - 1.486) | 0.799 (0.530 - 1.204) | 0.861 (0.571 - 1.298) |
| Black Caribbean | 0.889 (0.692 - 1.141) | 0.867 (0.672 - 1.118) | 0.774 (0.599 - 1.000) | 0.718 (0.555 - 0.928) |
| Chinese | 0.846 (0.526 - 1.363) | 0.845 (0.525 - 1.361) | 0.861 (0.534 - 1.388) | 1.041 (0.645 - 1.681) |
| Indian | 1.630 (1.400 - 1.896) | 1.580 (1.352 - 1.847) | 1.507 (1.280 - 1.773) | 1.460 (1.238 - 1.723) |
| Mixed | 1.179 (0.845 - 1.644) | 1.251 (0.896 - 1.747) | 1.131 (0.810 - 1.580) | 1.091 (0.781 - 1.525) |
| Other | 1.208 (0.971 - 1.502) | 1.310 (1.049 - 1.637) | 1.153 (0.918 - 1.448) | 1.135 (0.903 - 1.426) |
| Pakistani | 4.619 (4.006 - 5.326) | 3.220 (2.780 - 3.731) | 2.337 (1.962 - 2.784) | 1.994 (1.672 - 2.378) |
| White other | 0.675 (0.582 - 0.784) | 0.726 (0.624 - 0.845) | 0.729 (0.626 - 0.849) | 0.725 (0.622 - 0.844) |

Note: Linked 2011 Census to HES, GDPPR and Mortality registration data; Results obtained from Cox-regression models. Geographical factors: dummies for region of residence, for urban/rural classification and second order polynomial of population density of Lower Super Output Area (LSOA). Socio-demographic characteristics include Index of Multiple Deprivation (IMD), household deprivation (see table note), household tenure, social grade, level of highest qualification, household size, multigenerational household, household with children, key worker type, key worker in the household, exposure to disease, proximity to others, household exposure to disease, household proximity to others. Pre-pandemic health include Body Mass Index (kg/m2) , Chronic kidney disease (CKD), Learning disability, Cancer and immunosuppression, other conditions (See Supplementary Tables A1 for more details).
